# Assessment of Extractability and Accuracy of Electronic Health Record Data for Joint Implant Registries

**DOI:** 10.1001/jamanetworkopen.2021.1728

**Published:** 2021-03-15

**Authors:** Nicholas J. Giori, John Radin, Alison Callahan, Jason A. Fries, Eni Halilaj, Christopher Ré, Scott L. Delp, Nigam H. Shah, Alex H. S. Harris

**Affiliations:** 1 Center for Innovation to Implementation, VA Palo Alto Health Care System, Palo Alto, California; 2 Department of Orthopedic Surgery, Stanford University, Stanford, California; 3 Center for Biomedical Informatics Research, Stanford University, Stanford, California; 4 Department of Computer Science, Stanford University, Stanford, California; 5 Department of Bioengineering, Stanford University, Stanford, California; 6 Department of Surgery, Stanford University, Stanford, California

## Abstract

**Importance:**

Implant registries provide valuable information on the performance of implants in a real-world setting, yet they have traditionally been expensive to establish and maintain. Electronic health records (EHRs) are widely used and may include the information needed to generate clinically meaningful reports similar to a formal implant registry.

**Objectives:**

To quantify the extractability and accuracy of registry-relevant data from the EHR and to assess the ability of these data to track trends in implant use and the durability of implants (hereafter referred to as implant survivorship), using data stored since 2000 in the EHR of the largest integrated health care system in the United States.

**Design, Setting, and Participants:**

Retrospective cohort study of a large EHR of veterans who had 45 351 total hip arthroplasty procedures in Veterans Health Administration hospitals from 2000 to 2017. Data analysis was performed from January 1, 2000, to December 31, 2017.

**Exposures:**

Total hip arthroplasty.

**Main Outcomes and Measures:**

Number of total hip arthroplasty procedures extracted from the EHR, trends in implant use, and relative survivorship of implants.

**Results:**

A total of 45 351 total hip arthroplasty procedures were identified from 2000 to 2017 with 192 805 implant parts. Data completeness improved over the time. After 2014, 85% of prosthetic heads, 91% of shells, 81% of stems, and 85% of liners used in the Veterans Health Administration health care system were identified by part number. Revision burden and trends in metal vs ceramic prosthetic femoral head use were found to reflect data from the American Joint Replacement Registry. Recalled implants were obvious negative outliers in implant survivorship using Kaplan-Meier curves.

**Conclusions and Relevance:**

Although loss to follow-up remains a challenge that requires additional attention to improve the quantitative nature of calculated implant survivorship, we conclude that data collected during routine clinical care and stored in the EHR of a large health system over 18 years were sufficient to provide clinically meaningful data on trends in implant use and to identify poor implants that were subsequently recalled. This automated approach was low cost and had no reporting burden. This low-cost, low-overhead method to assess implant use and performance within a large health care setting may be useful to internal quality assurance programs and, on a larger scale, to postmarket surveillance of implant performance.

## Key Points

**Question:**

Are the data in a large US electronic health record (EHR) complete and accurate enough to track trends in implant use and to assess the durability of implants (hereafter referred to as implant survivorship)?

**Findings:**

In this cohort study, EHR records of patients who had total hip arthroplasty in all Veterans Health Administration hospitals since 2000 were automatically reviewed using novel software; 80% to 95% of hip replacement components used since 2014 were accurately identified, trends in implant use matched known national trends, and known poor implants were found to be negative outliers.

**Meaning:**

Automated analysis of the EHR provides a low-cost, low-overhead method to assess implant use and performance.

## Teaser

This cohort study quantifies the extractability and accuracy of registry-relevant data from the electronic health record (EHR) and assesses the ability of these data to track trends in implant use and implant survivorship, using data stored since 2000 in the EHR of the largest integrated health care system in the United States.

## Introduction

Joint replacement registries serve to monitor implant use, complications, and failure and to support recalls and advisories.^1,2,3,4,5,6,7,8,9,10^ Implant registries are costly to establish and maintain.^6^ To our knowledge, no existing orthopedic implant registry relies entirely on electronic health record (EHR) data. Existing registries require at least some dedicated data entry in addition to infrastructure, security, space, and staff. Because registry data must be tracked for decades, costs are ongoing, and years of investment are commonly needed before the value of a registry can be realized.

The Veterans Health Administration (VHA) is the largest integrated health care system in the United States. Implants placed in the VHA are not followed up in a formal registry. The VHA was an early adopter of a national EHR.^11^ The infrastructure of a national EHR is maintained as a requirement for clinical care. However, as with other US health care systems, the VHA does not care for a captured patient population. To our knowledge, it is unknown whether the data contained in the VHA EHR are extractable and interpretable by automated means or whether extracted data would be accurate and complete enough to provide clinically meaningful information similar to what can be provided by a formal implant registry.

In this cohort study, we sought (1) to quantify the extractability and accuracy of registry-relevant procedural information from the VHA EHR when using automated means and assess sources of missing data and error and (2) to assess the ability of these data to track trends in implant use and determine the durability of implants (hereafter referred to as implant survivorship). As tests of data utility, we hypothesized that, by using the VHA EHR, we would find that ceramic prosthetic femoral head use surpassed metal femoral head use at a similar time as reported by the American Joint Replacement Registry (AJRR) and that 2 recalled implants in the VHA EHR would be obvious negative outliers in Kaplan-Meier survivorship.

## Methods

This retrospective cohort study of 37 205 patients receiving total hip arthroplasty (THA) at any VHA medical center from 2000 to 2017 was approved by the institutional review board of Stanford University. A waiver of informed consent was granted because the research involved no more than minimal risk to the participants because it involved materials that have been previously collected, the rights and welfare of the participates were not adversely affected because procedures were in place to protect confidentiality, the research would not affect the treatment of patients, and because the research could not be practicably carried out without the requested waiver. The Strengthening the Reporting of Observational Studies in Epidemiology (STROBE) reporting guideline was followed.^12^

### Techniques of Data Extraction

#### Identifying the Patient Cohort

Our data source was the VHA Corporate Data Warehouse database, the central repository of information comprising the VHA EHR. It was created in 2006 to aggregate information stored in the VHA’s distributed data repositories and is updated daily. Patients who had primary or revision THA from 2000 through 2017 were identified using *Current Procedural Terminology* codes (27130, 27132, 27134, 27137, and 27138). Data on date of surgery and numeric patient identifier were collected from structured records.

#### Identifying the Side of Surgery and Part Numbers

Surgery side was identified from free text populating the Corporate Data Warehouse records entitled “ScheduledProcedure” or “PrincipalPostOpDiagnosis” using custom regular expressions. Prosthesis part numbers occupied a free-text field entitled “ProsthesisModel.” Extraneous characters were removed from the contents of this record to create a “cleaned” part number.

#### Identifying the Prosthesis From the Part Number

The cleaned part number was mapped onto the US Food and Drug Administration Global Unique Device Identification Database (GUDID), which has information on implants marketed after 2013. We enriched the GUDID database by adding part numbers of old implants that were commonly used or of particular interest.

Mapping the part number in GUDID, we identified company name, model, size, and whether the part was a major component of the THA (shell, liner, stem, or prosthetic femoral head) or not (such as screw or hole plug). For one manufacturer, the information in the GUDID did not identify model, so we hard-coded this information.

#### Aggregating Parts

The first 6 digits of the part number were used to aggregate different-sized parts of the same model. This worked in many cases but not all. Adjustments were made and hard-coded as needed.

#### Identifying When Implants Were Removed

Knowing the patient, surgery date, side of surgery, and THA components placed, we determined whether patients had a subsequent THA or revision anywhere in the VHA system on the same side. We identified new THA parts placed at the time of revision. We assumed that if a part, such as a stem, was placed at the time of revision, the prior stem must have been removed. Revisions of revisions were similarly tracked. The *Current Procedural Terminology* codes for removal of THA (ie, codes 27090 and 27091) identified operations in which implants were removed.

#### Calculating Kaplan-Meier Curves

Kaplan-Meier curves were calculated for prostheses used at least 100 times. Time to failure was the interval from implantation to removal for each THA component. Time to censor was the interval from implantation to last follow-up in any VHA clinic or to death. The VHA uses the Social Security Death Index, which identifies death occurring in a VHA facility or elsewhere.

### Assessment of Data Extractability and Accuracy

We determined the annual number of primary and revision THA procedures and the number and percentage of major parts identified by part number each year. To assess accuracy, a THA surgeon (N.J.G.) reviewed 100 randomly selected THA operations. Surgery date, side of surgery, and part numbers were compared with data collected via automated means. Sources of error were identified. Accuracy, precision, recall, and F1 metrics for the complete process of implant part number extraction, GUDID mapping, and major THA part identification were calculated.

### Assessment of Data Utility

To determine whether the trends in implant use were realistic, we tracked metal and ceramic femoral head use and identified when ceramic femoral head use surpassed metal femoral head use. This information was compared with the information reported by the AJRR.^13^

We then determined whether Kaplan-Meier curves for recalled implants used in the VHA system could be identified as negative outliers compared with other implants. For this information, we compared the survivorship (ie, durability) of each shell and stem with at least 100 uses with the survivorship of other shells or other stems over the entire time interval. Finally, as an initial evaluation of relative implant survivorship, we categorized shells and stems as having survivorship that was better than, similar to, or worse than all other stems or shells in our data set.

### Statistical Analysis

The 95% CIs of a proportion were calculated using the Wilson score interval.^14^ Kaplan-Meier curves with 95% CIs were used determine implant survivorship (CamDavidsonPilon/lifelines, version 0.256; GitHub). These Kaplan-Meier curves were deemed to be significantly different when the 95% CIs ceased to overlap.

## Results

We identified 45 351 primary and revision THA procedures performed on 37 205 patients; 94.7% of patients were male, reflecting the overwhelmingly male VHA patient population. The mean (SD) age at surgery was 63.7 (10.1) years (range, 21-99 years).

### Assessment of Data Extractability and Quality

**Figure 1.**
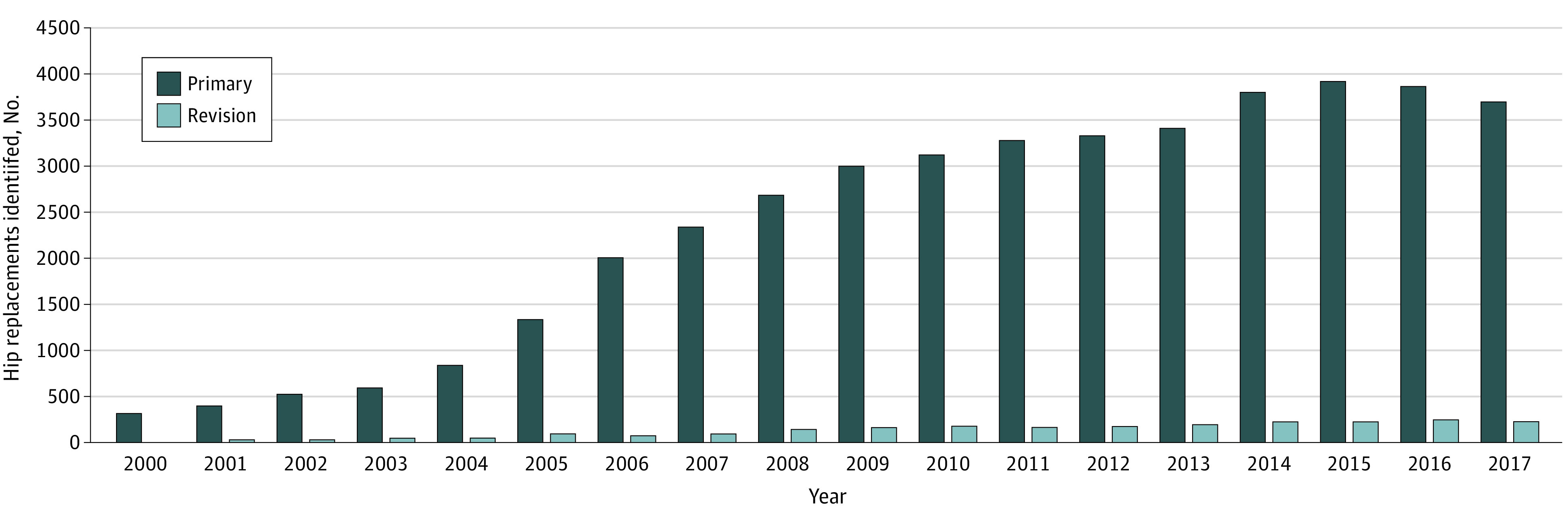
Data on Primary and Revision Total Hip Arthroplasty Procedures Identified by *Current Procedural Terminology* Codes in the Veterans Health Administration Corporate Data Warehouse Database

Primary and revision THA procedures increased over the course of the study (from 359 primary procedures and 0 revisions in 2000 to 3750 primary procedures and 214 revisions in 2017) (Figure 1). Revision burden (4.7% [2131 of 45 351]) was low compared with national registries with a captured patient population (approximately 10%)^15^ but was within the range of revision burden reported by the AJRR (4.0%-14.2%).^16^

**Figure 2.**
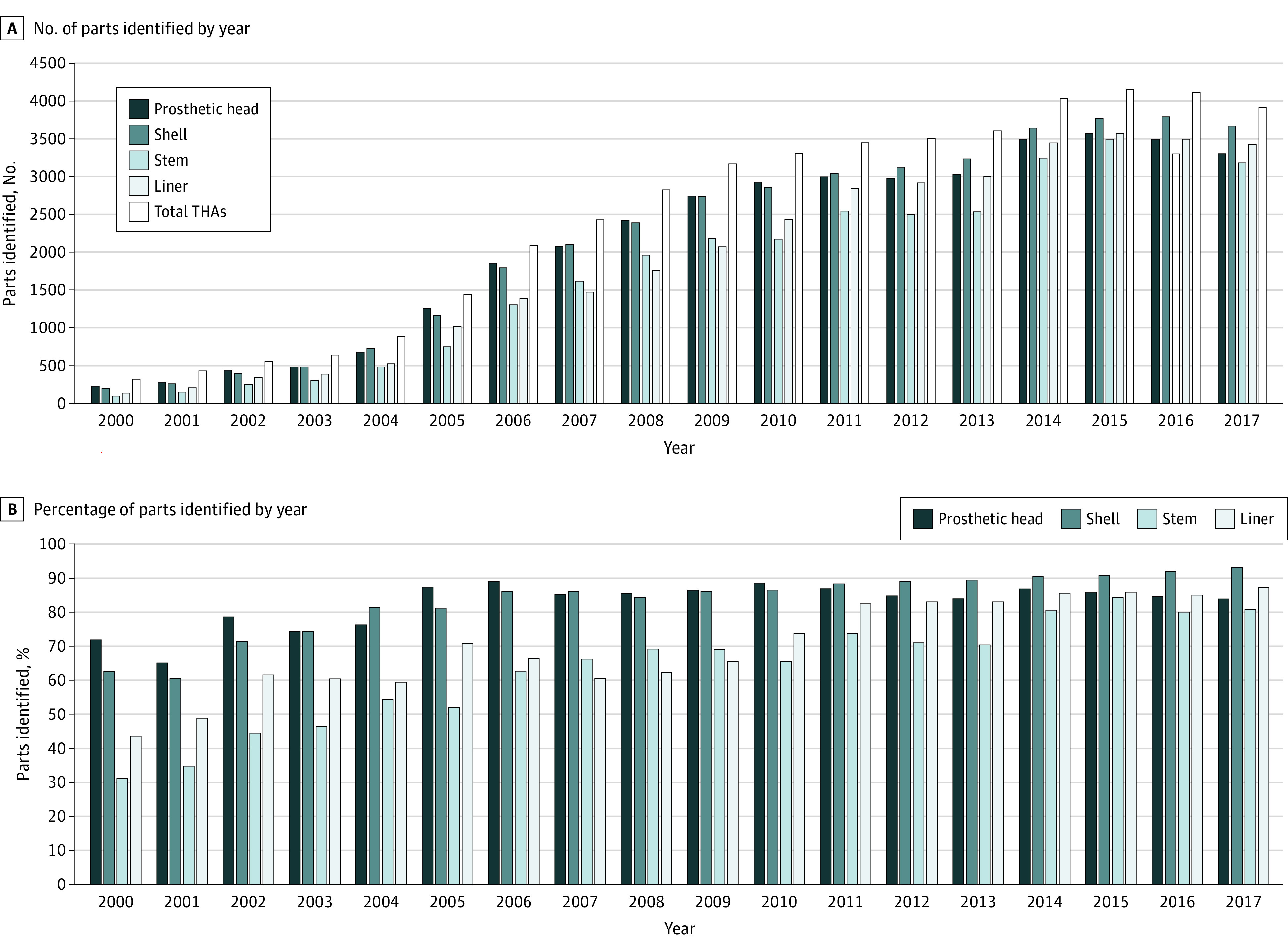
Data on Identified Primary and Revision Total Hip Arthroplasty Procedures (THAs) A, The total number of prosthetic femoral heads, shells, stems, and liners were identified by part number each year. B, The percentage of prosthetic femoral heads, shells, stems, and liners were identified by part number each year.

The number of major THA components identified each year is shown in Figure 2A. The percentage of major THA components identified each year is shown in Figure 2B. Since 2011, about 80% to 95% of shell, liner, and prosthetic femoral head parts were identified. Stem identification reached this range beginning in 2014. In comparison, the Australian Orthopedic Association National Joint Replacement Registry (AOANJRR) captures more than 97.8% of joint replacement procedures in Australia.^2^ The Kaiser Permanente Registry had 90% surgeon participation at the time it was established^8^; 98% of cases in the AJRR had acceptable implant part numbers submitted^9^ from an unknown fraction of THA cases in the United States.

The manual review of 100 randomly selected surgical procedures served to assess accuracy and completeness of automated data retrieval and sources of error (Table). Surgery side was identified correctly in all cases. Primary and revision procedures were differentiated correctly in 98 cases. All major implant part numbers were correctly identified in 75 surgical procedures, and they were correctly mapped to the GUDID database and fully identified in 51 surgical procedures.

**Table.** Results of Manual Medical Record Reviews From 100 Randomly Selected Surgical Procedures

	No. (% of total) [95% CI]^a^	Error log	Reason for error
Total No. of surgical cases reviewed	100		
Sex of patient present and identified	100 (100) [96-100]		
Date of birth present and identified	100 (100) [96-100]		
Date of surgery present and identified	100 (100) [96-100]		
Side of surgery identified correctly	100 (100) [96-100]		
*CPT* code differentiated primary from revision surgery correctly	98 (98) [93-99]		
Cases in which all part Nos. were correctly identified by algorithm	76 (76) [67-83]		
Cases in which all major implants were correctly identified by the algorithm	52 (52) [42-62]		
Total No. of major parts needing identification	391		
Accurate part No. found by manual review somewhere in record	352 (90) [87-93]	39	Missing data
Accurate part No. identified by computer	322 (82) [78-86]	14	Part No. entered into wrong record
		11	Excess characters not interpreted correctly
		5	Wrong No. entered
Part No. exists in GUDID database or augmented list	308 (79) [74-83]	14	Old part No.
Part No. mapped correctly in GUDID	288 (74) [69-78]	3	Upper vs lower case letter
		1	Part No. too short
		16	Uncertain problem
Major part type identified correctly in GMDN	283 (72) [68-77]	3	Synergy stem misclassified as a femoral head
		1	Ringloc shell misclassified as a ball
		1	Metal liner not identified as a liner

Abbreviations: *CPT*, *Current Procedural Terminology*; GMDN, Global Medical Device Nomenclature; GUDID, Global Unique Device Identification Database.

^a^ 95% CI calculated with the Wilson score interval.

The 100 cases had 391 major parts owing to partial revisions, monoblock shells, and a miscoded hemiarthroplasty. The reviewer found part numbers in the medical records for 352 major parts. The computer identified 322 correct part numbers; 308 part numbers existed in the augmented GUDID database, 288 part numbers were mapped successfully in the augmented GUDID, and 283 parts were ultimately fully described and correctly identified via automated means as one of the major parts of THA. Reasons for data loss and error are shown in the Table.

There were 517 major and minor parts in the 100 reviewed cases. We defined a true positive as when the part was fully and correctly identified as a major part, a true negative when the part was correctly identified as not a major part, a false positive as when identification as a major part was wrong, and a false negative as when the part was not identified as a major part. With these definitions, the manual review revealed 291 true positives, 116 true negatives, 5 false positives, and 105 false negatives in part identification. Accuracy was thus 79%, precision was 98%, recall was 73%, and F1 was 84%.

### Assessment of Data Utility

**Figure 3.**
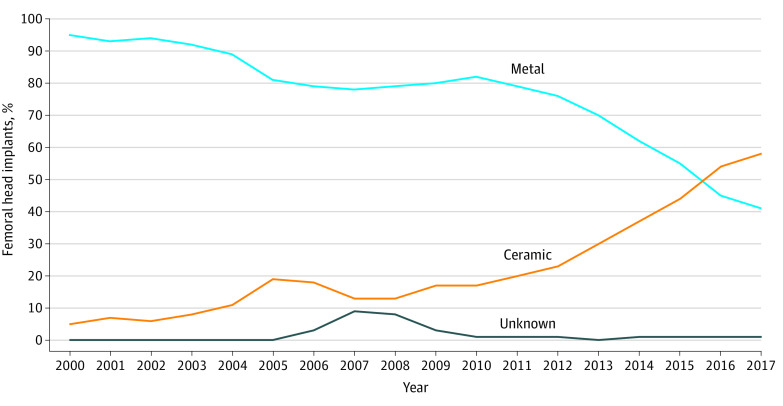
Data on Use of Cobalt Chrome and Ceramic Prosthetic Femoral Heads Shown as a Percentage of All Total Hip Arthroplasty Procedures Captured in the Corporate Data Warehouse Database The use of ceramic prosthetic femoral heads surpassed the use of cobalt chrome femoral heads between 2015 and 2016.

Metal and ceramic prosthetic femoral head use was tracked from 2000 to 2017 (Figure 3). Ceramic femoral head use surpassed metal femoral head use between 2015 (55% were metal femoral implant and 44% were ceramic femoral implant) and 2016 (45% were metal femoral implant and 54% were ceramic femoral implant). In the AJRR, metal was more common before 2015, and ceramic was more common after 2016. The AJRR reported roughly equal rates in 2015 and 2016.^13^

**Figure 4.**
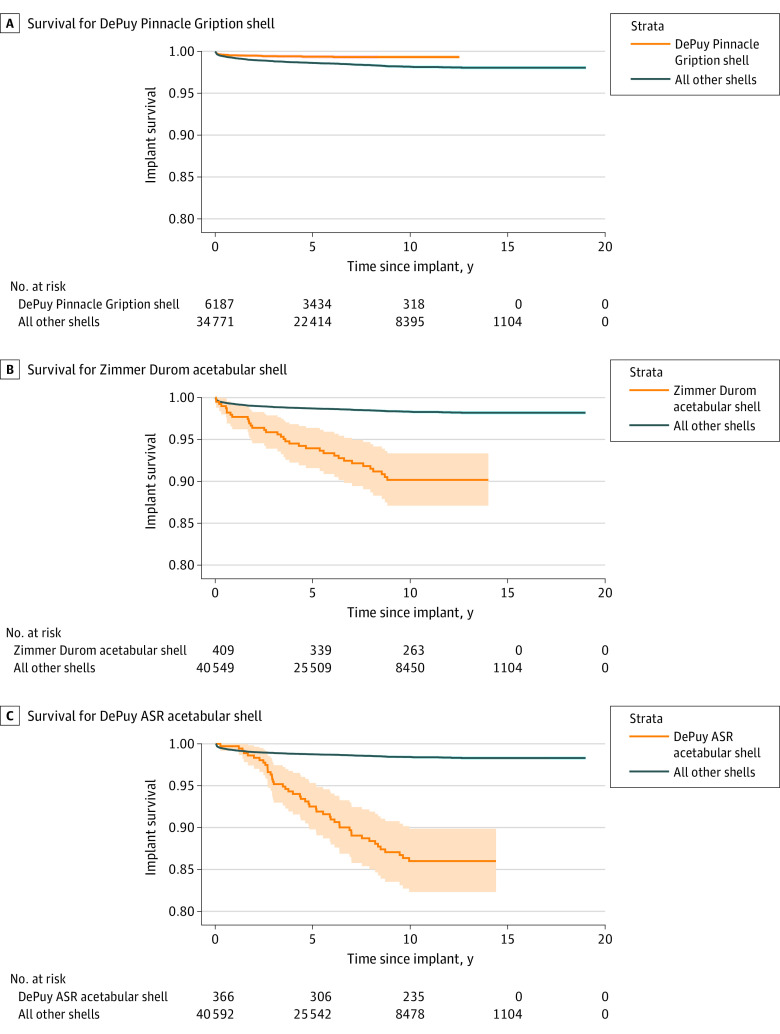
Kaplan-Meier Curve of Implant Survivorship A, This Kaplan-Meier curve shows an implant that was found to have better implant survivorship (ie, durability) than other shells in our cohort. The numbers ”at risk” indicate the numbers of implants that were still being tracked at each time point for the shell of interest and all the shells identified in the Veterans Health Administration (VHA) electronic health record (EHR). B and C, These Kaplan-Meier curves show the 2 worst implants that we could identify in the VHA EHR using automated techniques. Both implants were either recalled or withdrawn from the market and are no longer available.

We stratified individual implants as performing better than, similar to, or worse than other implants of the same type if their survivorship 95% CIs diverged and remained separated at all time points after diverging (eTables 1 and 2 in the Supplement). Implant-years of follow-up for each prosthesis were also calculated. We identified 6 high- and 3 low-survivorship acetabular shells. Figure 4 shows Kaplan-Meier curves for 3 shells in our study compared with the overall survivorship of other shells. The individual overall survivorship of all shells and stems in our study at 10 years was about 98%. The AOANJRR, in a captured patient population, reports a 10-year cumulative percent revision for primary THA for arthritis of 5% and a 10-year cumulative percent revision of all primary hip replacement (partial and total) for all diagnoses of about 7% (93%-95% 10-year survivorship).^2^

Figure 4 shows an example of a well-performing acetabular shell (Figure 4A) and the 2 worst-performing acetabular shells in our data set, the Zimmer Durom (Figure 4B) and the DePuy ASR (Figure 4C). Both of these implants have been found to have very poor survivorship in multiple registries as part of a nonhip resurfacing THA (ASR survivorship of 54%-57% at 10 years; Durom survivorship of 84%-85% at 10 years).^2,5^ Our quantitative 10-year survivorship results for the ASR and Durom of approximately 85% and 90%, respectively, are high compared with foreign national registries, which is consistent with all of our quantitative results. The Durom was withdrawn in 2008.^17^ The ASR was recalled in 2010.^18^ The third poorly performing shell had only 124 shells in the data set and is no longer marketed. No specific concerns regarding that shell had been previously raised in the literature. We identified 6 high-survivorship and 2 low-survivorship femoral stems (eTable 2 in the Supplement). Concern has been raised about 1 low-survivorship stem owing to high initial migration.^19^ The other low-surviving stem is used for revision surgery, explaining the low observed survivorship.

## Discussion

Electronic health records are designed to record individual episodes of patient care and facilitate billing. Data are entered for these purposes. It is thus reasonable to ask whether data in a large EHR are complete and accurate enough to be extracted and repurposed to track implant use and performance and whether this can be done on a large scale by automated means.

In our 100-case manual review, we quantified the extractability and accuracy of registry-relevant data from the VHA EHR and identified areas for improvement. Accuracy, precision, recall, and F1 for fully identifying major implant parts among the 517 total implant parts were 0.79, 0.98, 0.73, and 0.84, respectively. Because these metrics were based on a random sample of cases spanning the full study period, they represent a lower bound on quality metrics that could be expected in later years when data were more complete. Future data capture may improve as bar code scanner use increases and legacy implant use decreases. Developing code to extract part numbers from other locations in the EHR and adding other implant libraries to cross-reference part numbers may further improve performance.

Data extraction and quality were sufficient to identify the demographic characteristics of the patients and the trends in ceramic and metal femoral head use in the United States. Using a novel approach to identify when prosthesis components were explanted, we used 18 years of EHR data to generate Kaplan-Meier curves for individual THA prosthesis components.

Quantitative survivorship was overestimated compared with national registries with captured patient populations. As with all health systems in the United States, VHA patients with other insurance options^20^ may receive revision surgery elsewhere. Quantitative comparisons with other registries are difficult because our calculated survivorship is based on isolated implant component revision rates, whereas the AOANJRR and other national registries commonly report on implant pairs and define failure as revision for any reason, which may or may not include removal of the implant of interest. Assessment and improvement of quantitative results will require further analysis and likely incorporation of additional data sources, such as Medicare and community care as they become available. This is a subject for further work.

Relative implant survivorship, however, should be reliable if one assumes that the likelihood of patients going elsewhere for revision is unrelated to the implant model. This assumption is good enough to allow identification of the 2 recalled implants as obvious negative outliers. Implants with high survivorship (eTables 1 and 2 in the Supplement) had less survivorship deviation from other implants compared with the recalled implants (Figure 4). Four of the 5 high-survivorship shells and stems in our study with more than 10 000 implant-years of follow-up (DePuy Pinnacle and S&N R3 shells, and 2 DePuy Summit stems) are recognized in the AOANJRR as “superior” when paired with other good implants, but the fifth (Zimmer Continuum shell) is identified in the AOANJRR as having a higher than anticipated rate of revision. It is not necessarily expected that survivorship results will be consistent across registries given differences in practice patterns and patient demographic characteristics. Various factors, not simply implant design, may be associated with survivorship. Implants that we identify as high or low survivorship merit further investigation.

Identifying reasons for implant failure will be important for future development. *Current Procedural Terminology* codes for removal of all THA components (ie, codes 27090 and 27091) may identify failure due to periprosthetic infection. Other revisions should be due to aseptic failure. Natural language processing of the operative report may ultimately distinguish revision due to aseptic loosening, recurrent instability, or other aseptic processes.

Although this work begins the process of establishing a mature implant registry for the VHA, it is important to recognize the opportunity to further leverage the rich and diverse EHR data to track implant performance in novel ways. “Level 2” data, such as comorbidities, are difficult to capture in traditional registries^9^ but are easily captured with the EHR as the data source. This will facilitate analyses of implant failure that account for confounding factors, such as patient demographics, comorbidities, and socioeconomic status. Furthermore, different metrics of implant failure using novel inputs that are in the EHR but not routinely collected in other implant registries can be investigated.^21^ Analysis of unstructured notes with natural language processing or radiographs with machine learning^22,23,24,25,26,27^ may augment traditional means of implant surveillance and facilitate earlier and more sensitive detection of poor implant performance. These are innovations that we believe will contribute to quality enhancement efforts and improve the science of implant assessment and surveillance.

### Limitations

There were some limitations to this study. First, we were unable to identify patients who left the VHA system for revision surgery. Although it will be difficult to completely rectify this problem without extensive data sharing agreements among US health care professionals, data sources from Medicare and VHA community care are becoming available and may be used to improve overall data capture.

Second, information to notify veterans of implant recalls or advisories is not available if the primary surgery was outside of the VHA. Third, the veteran patient population is overwhelmingly male. This will normalize slightly with time as younger female veterans age, but the imbalance will remain substantial. Information gained will be useful for internal VHA quality initiatives, but it is not known whether relative implant survivorship results observed in this work are applicable beyond the VHA.

Fourth, we used the VHA EHR, which will be phased out as VHA converts to a commercial EHR. Commercial EHRs, such as EPIC and Cerner, have records for all of the relevant data fields that were drawn on to perform this analysis, including part number. With equivalent data entry in a commercial EHR, our overall approach should remain applicable.

Finally, this work was performed specifically for THA implants. However, all implant types have searchable part numbers in the GUDID database. Implantation information should thus be trackable for other implant types. If failure of another implant type is treated by replacement with a similar implant, then failure of these implantable prostheses should be similarly trackable. Obviously, the specifics of the method would need to be tailored to the implant of interest and validated.

## Conclusions

In this cohort study, extraction of registry-relevant information from the EHR of the largest integrated health care system in the United States was possible using automated means. The quantity and quality of the extracted data were sufficient to track trends in 18-year implant use and identify recalled implants as negative outliers. This approach was low cost and leveraged, in a novel way, the computational infrastructure of the EHR without adding reporting burden to hospital staff. The general approach that we describe may be applicable to the analysis of other implants in other large EHRs. To facilitate further development, our computer code is freely available in github^28^ and on request from the authors.

## Notes

Supplement. **eTable 1.** Assessment of Shell Survivorship **eTable 2.** Assessment of Stem Survivorship
