# Supplementary material for: Assessment of Extractability and Accuracy of Electronic Health Record Data for Joint Implant Registries
Source: JAMA Netw Open. 2021 Mar 15;4(3):e211728. doi: 10.1001/jamanetworkopen.2021.1728 (PMC7961313; doi:10.1001/jamanetworkopen.2021.1728)
Supplement: Supplement. — eTable 1. Assessment of Shell Survivorship eTable 2. Assessment of Stem Survivorship [file jamanetwopen-e211728-s001.pdf]

Supplemental Online Content

Giori NJ, Radin J, Callahan A, et al. Assessment of extractability and accuracy of electronic health record data for joint implant registries. *JAMA Netw Open*. 2021;4(3):e211728. doi:10.1001/jamanetworkopen.2021.1728

eTable 1. Assessment of Shell Survivorship

eTable 2. Assessment of Stem Survivorship

This supplemental material has been provided by the authors to give readers additional information about their work.

© 2021 Giori NJ et al. *JAMA Network Open.*

eTable 1. Assessment of Shell Survivorship				
Survivorship vs. All Other Shells	Shells	Number of Implants	Mean Implant-Years	Total Implant-Years
Better	DePuy Pinnacle Gription	6187	5.62	34770.94
	Zimmer Continuum	5079	5.50	27934.50
	S&N R3	3964	5.75	22793.00
	DePuy Duraloc	133	11.88	1580.04
	Biomet Ringloc	240	5.35	1284.00
	Exactech Novation	157	4.36	684.52
Similar	DePuy Pinnacle Porocoat	6023	7.95	47882.85
	Zimmer TM	3929	7.81	30685.49
	Zimmer Trilogy	3022	8.70	26291.40
	S&N Reflection	1558	9.48	14769.84
	Howmedica Tritanium	2210	4.67	10320.70
	Zimmer Converge	817	9.43	7704.31
	Howmedica Trident	655	6.32	4139.60
	Biomet Hip System	589	4.66	2744.74
	Howmedica SecurFit	231	7.99	1845.69
	Biomet M2A Magnum	179	9.37	1677.23
	Howmedica MDM	335	4.93	1651.55
	Biomet Regenerex Ringloc +	216	6.24	1347.84
	Biomet Ringloc +	227	5.92	1343.84
	Biomet G7 PPS	400	3.32	1328.00
	S&N Reflection HA	138	9.55	1317.90
	Exactech Accumatch A	114	11.49	1309.86
	Biomet G7 OsseoTi	374	2.91	1088.34
Worse	Zimmer Durom	409	9.62	3934.58
	DePuy ASR	366	9.67	3539.22
	Zimmer Trilogy Calcicoat	124	10.06	1247.44

© 2021 Giori NJ et al. *JAMA Network Open.*

**eTable 2. Assessment of Stem Survivorship**				
Survivorship vs. All Other Stems	Stems	Number of Implants	Mean Implant-Years	Total Implant-Years
Better	DePuy Summit Porocoat	4511	6.84	30855.24
	DePuy Summit DuoFix HA	2613	6.56	17141.28
	Zimmer ML Taper	1671	3.91	6533.61
	Zimmer Fiber Metal Midcoat Collared	337	9.29	3130.73
	Zimmer Fiber Metal Midcoat Collared HA	256	9.14	2339.84
	Exactech Accumatch P	146	8.72	1273.12
Even	Zimmer Kinectiv	1924	6.38	12275.12
	Zimmer TM Stem	1532	7.45	11413.40
	S&N Synergy	1383	7.63	10552.29
	S&N Synergy Porous plus HA	1102	7.04	7758.08
	S&N Anthology	1119	6.17	6904.23
	DePuy S-ROM	684	9.05	6190.20
	Howmedica Accolade II	1216	4.30	5228.80
	DePuy Corail	986	5.28	5206.08
	DePuy AML	494	6.92	3418.48
	Zimmer Kinectiv HA	540	6.00	3240.00
	Zimmer Fiber Metal Taper HA	256	10.09	2583.04
	S&N Echelon	225	5.81	1307.25
	Biomet Taperloc	119	9.28	1104.32
	DePuy Tri-Lock Gription	159	5.86	931.74
	Howmedica Accolade TMZF	111	7.83	869.13
	Zimmer Fitmore Stem	138	5.98	825.24
	Zimmer Fiber Metal Midcoat Collarless	104	7.79	810.16
	Zimmer Avenir	205	3.80	779.00
	Howmedica Restoration Modular	121	4.95	598.95
Worse	Zimmer Fiber Metal Taper	1242	8.96	11128.32
	Zimmer Revision Uncoated Stem	248	7.10	1760.80

© 2021 Giori NJ et al. *JAMA Network Open.*
